# Aqua­(4-fluoro­benzoato-κ*O*)bis­(1,10-phenanthroline-κ^2^ *N*,*N*′)manganese(II) 4-fluoro­benzoate trihydrate

**DOI:** 10.1107/S1600536811049968

**Published:** 2011-11-30

**Authors:** Yun-Xia Li, Bi-Song Zhang, Chang-Sheng Wu, Miao Zheng, Jian-Li Lin

**Affiliations:** a College of Pharmaceutics and Material Engineering, Jinhua College of Profession and Technology, Jinhua, Zhejiang 321007, People’s Republic of China; b State Key Laboratory Base of Novel Functional Materials and Preparation, Science Center of Applied Solid State Chemistry Research, Ningbo University, Ningbo, Zhejiang 315211, People’s Republic of China

## Abstract

In the title compound, [Mn(C_7_H_4_FO_2_)(C_12_H_8_N_2_)_2_(H_2_O)](C_7_H_4_FO_2_)·3H_2_O, the Mn^II^ atom is coordinated by four N atoms from two chelating 1,10-phenanthroline ligands and two O atoms from one monodentate 4-fluoro­benzoate ion and one water mol­ecule, forming a distorted octa­hedral geometry. In the crystal, the three components are assembled into a tape structure along the *a* axis by O—H⋯O and C—H⋯O hydrogen bonds. Between the tapes, a π–π inter­action with a centroid–centroid distance of 3.569 (3) Å and a weak C—H⋯F hydrogen bond are observed.

## Related literature

For applications of manganese complexes, see: Sehlotho & Durmus (2008 ▶). For related manganese(II) complexes with 1,10-phenanthroline ligands, see: Su *et al.* (2005 ▶); Zhang (2004 ▶).
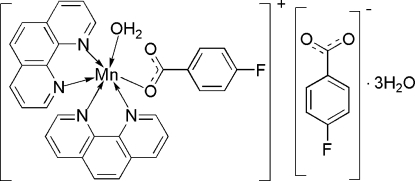


## Experimental

### 

#### Crystal data

[Mn(C_7_H_4_FO_2_)(C_12_H_8_N_2_)_2_(H_2_O)](C_7_H_4_FO_2_)·3H_2_O
*M* *_r_* = 765.62
Triclinic, 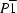

*a* = 8.8897 (17) Å
*b* = 14.773 (3) Å
*c* = 14.890 (3) Å
α = 107.815 (4)°
β = 107.314 (4)°
γ = 91.386 (4)°
*V* = 1762.9 (6) Å^3^
*Z* = 2
Mo *K*α radiation
μ = 0.45 mm^−1^
*T* = 290 K
0.20 × 0.15 × 0.12 mm

#### Data collection

Bruker SMART APEX CCD diffractometer
Absorption correction: multi-scan (*SADABS*; Bruker, 2000 ▶) *T*_min_ = 0.923, *T*_max_ = 0.948
9353 measured reflections
6138 independent reflections
4642 reflections with *I* > 2σ(*I*)
*R*_int_ = 0.032

#### Refinement

*R*[*F* ^2^ > 2σ(*F* ^2^)] = 0.085
*wR*(*F* ^2^) = 0.178
*S* = 1.14
6138 reflections
478 parameters
H-atom parameters constrained
Δρ_max_ = 0.38 e Å^−3^
Δρ_min_ = −0.26 e Å^−3^

### 

Data collection: *SMART* (Bruker, 1998 ▶); cell refinement: *SAINT* (Bruker, 1998 ▶); data reduction: *SAINT*; program(s) used to solve structure: *SHELXS97* (Sheldrick, 2008 ▶); program(s) used to refine structure: *SHELXL97* (Sheldrick, 2008 ▶); molecular graphics: *ORTEPII* (Johnson, 1976 ▶) and *DIAMOND* (Brandenburg & Putz, 1999 ▶); software used to prepare material for publication: *SHELXL97*.

## Supplementary Material

Crystal structure: contains datablock(s) global, I. DOI: 10.1107/S1600536811049968/is2782sup1.cif

Structure factors: contains datablock(s) I. DOI: 10.1107/S1600536811049968/is2782Isup2.hkl

Additional supplementary materials:  crystallographic information; 3D view; checkCIF report

## Figures and Tables

**Table 1:** Hydrogen-bond geometry (Å, °)

*D*—H⋯*A*	*D*—H	H⋯*A*	*D*⋯*A*	*D*—H⋯*A*
O5—H5*A*⋯O3^i^	0.85	1.79	2.622 (4)	166
O5—H5*B*⋯O2	0.85	2.06	2.719 (5)	135
O6—H6*A*⋯O4^ii^	0.85	1.98	2.825 (6)	171
O6—H6*B*⋯O7^iii^	0.85	2.08	2.827 (7)	146
O7—H7*A*⋯O8	0.85	2.02	2.854 (8)	165
O7—H7*B*⋯O6	0.85	1.99	2.819 (6)	166
O8—H8*A*⋯O4	0.85	1.97	2.792 (7)	164
C1—H1⋯F2^iv^	0.93	2.50	3.209 (7)	133
C5—H5⋯O3^v^	0.93	2.45	3.339 (7)	160
C20—H20⋯O4^i^	0.93	2.42	3.233 (7)	146

Symmetry codes: (i) 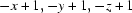; (ii) 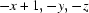; (iii) 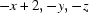; (iv) 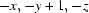; (v) 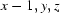.

**Fig. 1.**
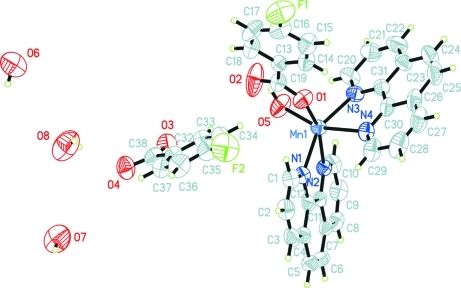
The molecular structure of the title compound, showing the atom-labelling scheme. Displacement ellipsoids are drawn at the 50% probability level.

**Fig. 2.**
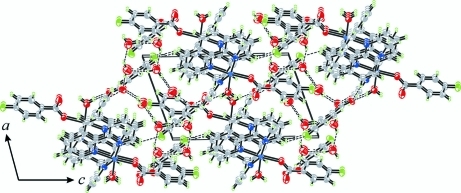
The three-dimensional supramolecular network of the title complex. Hydrogen bonds are drawn as dashed lines. H atoms not involved in the hydrogen bonds have been omitted.

## supplementary crystallographic information

### Comment

Potential applications of manganese complexes have been reflected in catalysis, molecular magnets, materials, biology, electrochemical properties, *etc* (Sehlotho & Durmus, 2008). In this paper, we report synthesis and structure of a new manganese coordination complex with 4-fluorobenzoic acid, 1,10-phenanthroline and water ligands. The crystal structure of title compound is similar to the reported structures (Su *et al.*, 2005; Zhang, 2004). In the complex molecule, the Mn^II^ atom is coordinated by four N atoms from two phen ligands, two O atoms respectively from one 4-fluorobenzoate ion and one water molecule to form a distorted MnN_4_O_2_ octahedral geometry. The equatorial positions of the Mn^II^ ion are occupied by one carboxylate O atom from the 4-fluorobenzoate ion and three N atoms from different phen molecules, and the axial ones by the other N atom from one phen ligand and one carboxylate O atom from one water molecule. The Mn1—N bond length is 2.245 (4) to 2.338 (4) Å, and Mn1—O bond lengths are 2.100 (3) and 2.126 (3) Å (Fig. 1). In the crystal structure, a tape structure of the three components along the *a* direction is formed by O—H···O and C—H···O hydrogen bonds (Table 1 and Fig. 2). A π–π stacking interaction between two adjacent phen ligands, with an interplanar distance of 3.389 (2) Å and a centroid-centroid distance of 3.569 (3) Å, and a weak C—H···F interaction are observed between the tapes.

### Experimental

MnCl_2_.2H_2_O (0.081 g, 0.50 mmol) was dissolved in appropriate amount of water, and then 1*M* Na_2_CO_3_ solution was added. MnCO_3_ was obtained by filtration, which was then washed with distilled water for 5 times. The freshly prepared MnCO_3_, 4-fluorobenzoic acid (0.070 g, 0.50 mmol), phen.H_2_O (0.099 g, 0.50 mmol), CH_3_OH/H_2_O (*v*/*v* = 1:2, 15 ml) were mixed and stirred for 6 h. Subsequently, the resulting cream suspension was heated in a 23 ml Teflon-lined stainless steel autoclave at 453 K for ca. 260 h. After the autoclave was cooled to room temperature, the solid was filtered off. The resulting filtrate was allowed to stand at room temperature, and slow evaporation for a week afforded yellow bulk single crystals.

### Refinement

C-bound H atoms were placed in calculated positions (C—H = 0.93 Å) and were refined using the riding-model approximation, with *U*_iso_(H) = 1.2*U*_eq_(C). H atoms attached to O atoms were found in a difference Fourier map and were refined using a riding model, with the O—H distances fixed as initially found, and with *U*_iso_(H) = 1.5*U*_eq_(O).

### Crystal data

**Table:**

[Mn(C_7_H_4_FO_2_)(C_12_H_8_N_2_)_2_(H_2_O)](C_7_H_4_FO_2_)·3H_2_O	*Z* = 2
*M**_r_* = 765.62	*F*(000) = 790
Triclinic, *P*1	*D*_x_ = 1.442 Mg m^−^^3^
Hall symbol: -P 1	Mo *K*α radiation, λ = 0.71073 Å
*a* = 8.8897 (17) Å	Cell parameters from 6392 reflections
*b* = 14.773 (3) Å	θ = 1.7–25.0°
*c* = 14.890 (3) Å	µ = 0.45 mm^−^^1^
α = 107.815 (4)°	*T* = 290 K
β = 107.314 (4)°	Block, yellow
γ = 91.386 (4)°	0.20 × 0.15 × 0.12 mm
*V* = 1762.9 (6) Å^3^	

### Data collection

**Table:**

Bruker SMART APEX CCD diffractometer	6138 independent reflections
Radiation source: fine-focus sealed tube	4642 reflections with *I* > 2σ(*I*)
graphite	*R*_int_ = 0.032
Detector resolution: 0 pixels mm^-1^	θ_max_ = 25.0°, θ_min_ = 1.7°
φ and ω scans	*h* = −10→8
Absorption correction: multi-scan (*SADABS*; Bruker, 2000)	*k* = −16→17
*T*_min_ = 0.923, *T*_max_ = 0.948	*l* = −17→17
9353 measured reflections	

### Refinement

**Table:**

Refinement on *F*^2^	Primary atom site location: structure-invariant direct methods
Least-squares matrix: full	Secondary atom site location: difference Fourier map
*R*[*F*^2^ > 2σ(*F*^2^)] = 0.085	Hydrogen site location: inferred from neighbouring sites
*wR*(*F*^2^) = 0.178	H-atom parameters constrained
*S* = 1.14	*w* = 1/[σ^2^(*F*_o_^2^) + (0.0644*P*)^2^ + 0.7608*P*] where *P* = (*F*_o_^2^ + 2*F*_c_^2^)/3
6138 reflections	(Δ/σ)_max_ < 0.001
478 parameters	Δρ_max_ = 0.38 e Å^−^^3^
0 restraints	Δρ_min_ = −0.26 e Å^−^^3^

### Special details

**Table:**

Geometry. All e.s.d.'s (except the e.s.d. in the dihedral angle between two l.s. planes) are estimated using the full covariance matrix. The cell e.s.d.'s are taken into account individually in the estimation of e.s.d.'s in distances, angles and torsion angles; correlations between e.s.d.'s in cell parameters are only used when they are defined by crystal symmetry. An approximate (isotropic) treatment of cell e.s.d.'s is used for estimating e.s.d.'s involving l.s. planes.
Refinement. Refinement of *F*^2^ against ALL reflections. The weighted *R*-factor *wR* and goodness of fit *S* are based on *F*^2^, conventional *R*-factors *R* are based on *F*, with *F* set to zero for negative *F*^2^. The threshold expression of *F*^2^ > σ(*F*^2^) is used only for calculating *R*-factors(gt) *etc*. and is not relevant to the choice of reflections for refinement. *R*-factors based on *F*^2^ are statistically about twice as large as those based on *F*, and *R*- factors based on ALL data will be even larger.

### Fractional atomic coordinates and isotropic or equivalent isotropic displacement parameters (Å^2^)

**Table:**

	*x*	*y*	*z*	*U*_iso_*/*U*_eq_	
Mn1	0.21473 (8)	0.73431 (5)	0.44141 (5)	0.0408 (2)	
N1	0.0199 (4)	0.6106 (3)	0.3641 (3)	0.0436 (9)	
N2	0.1238 (4)	0.7091 (2)	0.5605 (3)	0.0412 (9)	
N3	0.3123 (4)	0.8885 (3)	0.5313 (3)	0.0483 (10)	
N4	0.0374 (5)	0.8306 (3)	0.3773 (3)	0.0491 (10)	
O1	0.2786 (4)	0.7154 (3)	0.3125 (2)	0.0580 (9)	
O2	0.4787 (6)	0.6302 (3)	0.3210 (3)	0.1050 (16)	
O3	0.4419 (4)	0.3208 (3)	0.3175 (2)	0.0703 (10)	
O4	0.3937 (4)	0.1706 (3)	0.2153 (3)	0.0715 (10)	
O5	0.4333 (4)	0.6861 (3)	0.5018 (2)	0.0717 (11)	
H5A	0.4864	0.6905	0.5611	0.107*	
H5B	0.4985	0.6815	0.4693	0.107*	
O6	0.8404 (5)	−0.0093 (3)	−0.1362 (3)	0.1048 (15)	
H6A	0.7712	−0.0585	−0.1659	0.157*	
H6B	0.9319	−0.0227	−0.1386	0.157*	
O7	0.8483 (6)	0.0538 (3)	0.0642 (3)	0.1200 (17)	
H7A	0.7639	0.0614	0.0799	0.180*	
H7B	0.8283	0.0331	0.0015	0.180*	
O8	0.5651 (6)	0.0442 (4)	0.1157 (4)	0.140 (2)	
H8A	0.5085	0.0864	0.1360	0.211*	
H8B	0.5529	−0.0061	0.1304	0.211*	
F1	0.4141 (4)	0.7201 (3)	−0.0743 (2)	0.1016 (12)	
F2	0.0334 (5)	0.3861 (3)	−0.0739 (3)	0.1139 (14)	
C1	−0.0280 (6)	0.5602 (3)	0.2679 (4)	0.0549 (13)	
H1	0.0316	0.5697	0.2290	0.066*	
C2	−0.1629 (6)	0.4939 (4)	0.2231 (4)	0.0626 (14)	
H2	−0.1940	0.4610	0.1551	0.075*	
C3	−0.2494 (6)	0.4769 (3)	0.2781 (4)	0.0609 (14)	
H3	−0.3395	0.4318	0.2481	0.073*	
C4	−0.2039 (5)	0.5272 (3)	0.3804 (4)	0.0480 (12)	
C5	−0.2856 (6)	0.5127 (4)	0.4450 (4)	0.0603 (14)	
H5	−0.3770	0.4687	0.4188	0.072*	
C6	−0.2330 (6)	0.5614 (4)	0.5425 (4)	0.0596 (14)	
H6	−0.2890	0.5507	0.5829	0.071*	
C7	−0.0926 (5)	0.6297 (3)	0.5865 (4)	0.0465 (11)	
C8	−0.0295 (6)	0.6806 (4)	0.6890 (4)	0.0578 (14)	
H8	−0.0818	0.6729	0.7323	0.069*	
C9	0.1071 (7)	0.7407 (4)	0.7244 (4)	0.0600 (14)	
H9	0.1516	0.7730	0.7923	0.072*	
C10	0.1804 (6)	0.7539 (3)	0.6585 (3)	0.0526 (12)	
H10	0.2739	0.7960	0.6839	0.063*	
C11	−0.0099 (5)	0.6471 (3)	0.5256 (3)	0.0402 (10)	
C12	−0.0656 (5)	0.5940 (3)	0.4209 (3)	0.0393 (10)	
C13	0.3921 (5)	0.6878 (3)	0.1850 (3)	0.0433 (11)	
C14	0.3068 (6)	0.7495 (3)	0.1460 (3)	0.0549 (13)	
H14	0.2419	0.7841	0.1789	0.066*	
C15	0.3127 (6)	0.7629 (4)	0.0591 (4)	0.0673 (15)	
H15	0.2543	0.8059	0.0336	0.081*	
C16	0.4077 (7)	0.7102 (4)	0.0129 (4)	0.0649 (15)	
C17	0.4943 (7)	0.6478 (4)	0.0472 (4)	0.0727 (16)	
H17	0.5569	0.6127	0.0125	0.087*	
C18	0.4889 (6)	0.6365 (4)	0.1358 (4)	0.0655 (15)	
H18	0.5500	0.5946	0.1617	0.079*	
C19	0.3842 (6)	0.6751 (3)	0.2804 (3)	0.0499 (12)	
C20	0.4439 (6)	0.9168 (4)	0.6097 (4)	0.0598 (14)	
H20	0.4912	0.8711	0.6361	0.072*	
C21	0.5130 (7)	1.0104 (4)	0.6534 (4)	0.0674 (15)	
H21	0.6019	1.0276	0.7101	0.081*	
C22	0.4513 (7)	1.0775 (4)	0.6136 (4)	0.0737 (17)	
H22	0.5005	1.1405	0.6409	0.088*	
C23	0.3127 (7)	1.0518 (4)	0.5310 (4)	0.0599 (14)	
C24	0.2387 (8)	1.1156 (4)	0.4826 (5)	0.0799 (18)	
H24	0.2836	1.1793	0.5065	0.096*	
C25	0.1081 (9)	1.0878 (4)	0.4048 (5)	0.0797 (18)	
H25	0.0650	1.1320	0.3746	0.096*	
C26	0.0309 (7)	0.9910 (4)	0.3656 (4)	0.0619 (14)	
C27	−0.1097 (8)	0.9586 (5)	0.2863 (4)	0.0785 (18)	
H27	−0.1585	1.0003	0.2543	0.094*	
C28	−0.1755 (7)	0.8665 (5)	0.2557 (4)	0.0795 (18)	
H28	−0.2714	0.8447	0.2042	0.095*	
C29	−0.0970 (6)	0.8045 (4)	0.3028 (4)	0.0669 (15)	
H29	−0.1424	0.7409	0.2803	0.080*	
C30	0.1009 (6)	0.9242 (3)	0.4099 (3)	0.0471 (12)	
C31	0.2448 (6)	0.9546 (3)	0.4926 (3)	0.0472 (12)	
C32	0.2864 (5)	0.2925 (3)	0.1510 (3)	0.0452 (11)	
C33	0.2816 (5)	0.3889 (4)	0.1659 (3)	0.0501 (12)	
H33	0.3369	0.4330	0.2279	0.060*	
C34	0.1962 (6)	0.4213 (4)	0.0906 (4)	0.0611 (14)	
H34	0.1937	0.4865	0.1007	0.073*	
C35	0.1162 (7)	0.3552 (5)	0.0016 (4)	0.0698 (16)	
C36	0.1153 (7)	0.2590 (5)	−0.0166 (4)	0.0738 (17)	
H36	0.0571	0.2157	−0.0784	0.089*	
C37	0.2029 (6)	0.2273 (4)	0.0589 (3)	0.0585 (13)	
H37	0.2057	0.1620	0.0478	0.070*	
C38	0.3824 (6)	0.2584 (4)	0.2351 (4)	0.0524 (12)	

### Atomic displacement parameters (Å^2^)

**Table:**

	*U*^11^	*U*^22^	*U*^33^	*U*^12^	*U*^13^	*U*^23^
Mn1	0.0408 (4)	0.0452 (4)	0.0389 (4)	0.0043 (3)	0.0097 (3)	0.0202 (3)
N1	0.047 (2)	0.046 (2)	0.036 (2)	0.0069 (18)	0.0086 (18)	0.0159 (18)
N2	0.041 (2)	0.042 (2)	0.040 (2)	0.0063 (17)	0.0106 (18)	0.0159 (17)
N3	0.049 (2)	0.051 (2)	0.045 (2)	0.0000 (19)	0.013 (2)	0.0194 (19)
N4	0.051 (2)	0.055 (3)	0.040 (2)	0.0133 (19)	0.009 (2)	0.0176 (19)
O1	0.052 (2)	0.086 (3)	0.0476 (19)	0.0160 (19)	0.0186 (17)	0.0347 (18)
O2	0.145 (4)	0.131 (4)	0.076 (3)	0.097 (3)	0.052 (3)	0.063 (3)
O3	0.076 (3)	0.078 (3)	0.041 (2)	0.013 (2)	−0.0026 (19)	0.0177 (19)
O4	0.074 (3)	0.065 (3)	0.067 (2)	0.018 (2)	0.006 (2)	0.025 (2)
O5	0.060 (2)	0.112 (3)	0.059 (2)	0.037 (2)	0.0189 (18)	0.049 (2)
O6	0.104 (3)	0.099 (3)	0.100 (3)	0.000 (3)	0.015 (3)	0.036 (3)
O7	0.106 (4)	0.137 (4)	0.093 (3)	0.016 (3)	0.036 (3)	−0.001 (3)
O8	0.110 (4)	0.153 (5)	0.148 (5)	0.034 (4)	0.057 (4)	0.018 (4)
F1	0.102 (3)	0.160 (4)	0.058 (2)	0.009 (2)	0.038 (2)	0.045 (2)
F2	0.117 (3)	0.149 (4)	0.080 (2)	0.020 (3)	−0.002 (2)	0.076 (2)
C1	0.062 (3)	0.053 (3)	0.045 (3)	0.010 (3)	0.012 (3)	0.015 (2)
C2	0.062 (4)	0.056 (3)	0.048 (3)	0.008 (3)	−0.001 (3)	0.006 (3)
C3	0.049 (3)	0.043 (3)	0.077 (4)	0.003 (2)	0.004 (3)	0.015 (3)
C4	0.044 (3)	0.040 (3)	0.060 (3)	0.011 (2)	0.009 (2)	0.023 (2)
C5	0.043 (3)	0.060 (3)	0.089 (4)	0.009 (2)	0.020 (3)	0.040 (3)
C6	0.047 (3)	0.070 (4)	0.083 (4)	0.018 (3)	0.030 (3)	0.045 (3)
C7	0.046 (3)	0.052 (3)	0.058 (3)	0.024 (2)	0.024 (2)	0.033 (2)
C8	0.063 (4)	0.074 (4)	0.061 (3)	0.033 (3)	0.034 (3)	0.042 (3)
C9	0.070 (4)	0.072 (4)	0.043 (3)	0.020 (3)	0.017 (3)	0.027 (3)
C10	0.062 (3)	0.050 (3)	0.042 (3)	0.008 (2)	0.009 (2)	0.018 (2)
C11	0.042 (3)	0.039 (3)	0.049 (3)	0.016 (2)	0.015 (2)	0.028 (2)
C12	0.036 (2)	0.039 (3)	0.044 (3)	0.009 (2)	0.008 (2)	0.019 (2)
C13	0.045 (3)	0.042 (3)	0.036 (2)	0.003 (2)	0.012 (2)	0.006 (2)
C14	0.064 (3)	0.062 (3)	0.042 (3)	0.017 (3)	0.021 (3)	0.018 (2)
C15	0.067 (4)	0.086 (4)	0.057 (3)	0.023 (3)	0.019 (3)	0.034 (3)
C16	0.060 (4)	0.092 (4)	0.041 (3)	0.003 (3)	0.013 (3)	0.023 (3)
C17	0.057 (4)	0.106 (5)	0.054 (3)	0.020 (3)	0.030 (3)	0.012 (3)
C18	0.064 (4)	0.070 (4)	0.062 (3)	0.024 (3)	0.020 (3)	0.020 (3)
C19	0.050 (3)	0.051 (3)	0.045 (3)	0.009 (2)	0.013 (2)	0.013 (2)
C20	0.062 (3)	0.056 (3)	0.057 (3)	−0.007 (3)	0.011 (3)	0.023 (3)
C21	0.065 (4)	0.066 (4)	0.061 (3)	−0.005 (3)	0.012 (3)	0.015 (3)
C22	0.077 (4)	0.058 (4)	0.079 (4)	−0.017 (3)	0.034 (4)	0.005 (3)
C23	0.078 (4)	0.045 (3)	0.072 (4)	0.008 (3)	0.043 (3)	0.023 (3)
C24	0.098 (5)	0.050 (4)	0.111 (5)	0.018 (3)	0.050 (4)	0.037 (4)
C25	0.106 (5)	0.067 (4)	0.100 (5)	0.046 (4)	0.053 (4)	0.053 (4)
C26	0.076 (4)	0.070 (4)	0.060 (3)	0.037 (3)	0.033 (3)	0.035 (3)
C27	0.092 (5)	0.093 (5)	0.070 (4)	0.052 (4)	0.030 (4)	0.047 (4)
C28	0.071 (4)	0.110 (5)	0.050 (3)	0.039 (4)	0.005 (3)	0.028 (4)
C29	0.067 (4)	0.075 (4)	0.049 (3)	0.024 (3)	0.007 (3)	0.017 (3)
C30	0.059 (3)	0.056 (3)	0.040 (3)	0.026 (2)	0.026 (2)	0.024 (2)
C31	0.058 (3)	0.049 (3)	0.046 (3)	0.010 (2)	0.027 (2)	0.020 (2)
C32	0.036 (3)	0.062 (3)	0.038 (3)	0.007 (2)	0.012 (2)	0.016 (2)
C33	0.050 (3)	0.059 (3)	0.038 (3)	0.002 (2)	0.014 (2)	0.012 (2)
C34	0.060 (3)	0.071 (4)	0.061 (3)	0.012 (3)	0.017 (3)	0.036 (3)
C35	0.065 (4)	0.100 (5)	0.056 (4)	0.020 (3)	0.014 (3)	0.046 (4)
C36	0.069 (4)	0.101 (5)	0.037 (3)	0.012 (3)	0.002 (3)	0.017 (3)
C37	0.060 (3)	0.060 (3)	0.049 (3)	0.010 (3)	0.012 (3)	0.013 (3)
C38	0.040 (3)	0.071 (4)	0.045 (3)	0.008 (3)	0.009 (2)	0.021 (3)

### Geometric parameters (Å, °)

**Table:**

Mn1—O1	2.101 (3)	C9—H9	0.9300
Mn1—O5	2.123 (3)	C10—H10	0.9300
Mn1—N1	2.245 (4)	C11—C12	1.437 (6)
Mn1—N3	2.254 (4)	C13—C14	1.355 (6)
Mn1—N2	2.276 (3)	C13—C18	1.386 (7)
Mn1—N4	2.338 (4)	C13—C19	1.510 (6)
N1—C1	1.325 (5)	C14—C15	1.382 (6)
N1—C12	1.361 (5)	C14—H14	0.9300
N2—C10	1.333 (5)	C15—C16	1.358 (7)
N2—C11	1.346 (5)	C15—H15	0.9300
N3—C20	1.331 (6)	C16—C17	1.338 (8)
N3—C31	1.346 (6)	C17—C18	1.394 (7)
N4—C29	1.316 (6)	C17—H17	0.9300
N4—C30	1.362 (6)	C18—H18	0.9300
O1—C19	1.258 (5)	C20—C21	1.372 (7)
O2—C19	1.217 (6)	C20—H20	0.9300
O3—C38	1.238 (6)	C21—C22	1.352 (8)
O4—C38	1.251 (6)	C21—H21	0.9300
O5—H5A	0.8501	C22—C23	1.401 (7)
O5—H5B	0.8499	C22—H22	0.9300
O6—H6A	0.8500	C23—C31	1.415 (6)
O6—H6B	0.8499	C23—C24	1.416 (7)
O7—H7A	0.8501	C24—C25	1.321 (8)
O7—H7B	0.8498	C24—H24	0.9300
O8—H8A	0.8500	C25—C26	1.434 (8)
O8—H8B	0.8500	C25—H25	0.9300
F1—C16	1.368 (5)	C26—C27	1.390 (8)
F2—C35	1.360 (6)	C26—C30	1.408 (6)
C1—C2	1.382 (7)	C27—C28	1.350 (8)
C1—H1	0.9300	C27—H27	0.9300
C2—C3	1.348 (7)	C28—C29	1.397 (7)
C2—H2	0.9300	C28—H28	0.9300
C3—C4	1.400 (7)	C29—H29	0.9300
C3—H3	0.9300	C30—C31	1.432 (6)
C4—C12	1.410 (6)	C32—C33	1.376 (6)
C4—C5	1.425 (7)	C32—C37	1.382 (6)
C5—C6	1.337 (7)	C32—C38	1.524 (6)
C5—H5	0.9300	C33—C34	1.379 (6)
C6—C7	1.432 (7)	C33—H33	0.9300
C6—H6	0.9300	C34—C35	1.353 (7)
C7—C11	1.400 (6)	C34—H34	0.9300
C7—C8	1.406 (7)	C35—C36	1.361 (8)
C8—C9	1.350 (7)	C36—C37	1.382 (7)
C8—H8	0.9300	C36—H36	0.9300
C9—C10	1.384 (6)	C37—H37	0.9300
			
O1—Mn1—O5	87.22 (13)	C13—C14—H14	118.7
O1—Mn1—N1	92.61 (13)	C15—C14—H14	118.7
O5—Mn1—N1	110.41 (14)	C16—C15—C14	116.8 (5)
O1—Mn1—N3	102.43 (13)	C16—C15—H15	121.6
O5—Mn1—N3	91.99 (15)	C14—C15—H15	121.6
N1—Mn1—N3	153.68 (14)	C17—C16—C15	123.7 (5)
O1—Mn1—N2	163.65 (13)	C17—C16—F1	117.8 (5)
O5—Mn1—N2	90.34 (13)	C15—C16—F1	118.5 (5)
N1—Mn1—N2	73.11 (13)	C16—C17—C18	118.7 (5)
N3—Mn1—N2	93.81 (13)	C16—C17—H17	120.7
O1—Mn1—N4	83.87 (13)	C18—C17—H17	120.7
O5—Mn1—N4	159.63 (14)	C13—C18—C17	119.7 (5)
N1—Mn1—N4	88.31 (13)	C13—C18—H18	120.1
N3—Mn1—N4	72.22 (14)	C17—C18—H18	120.1
N2—Mn1—N4	103.18 (13)	O2—C19—O1	125.4 (5)
C1—N1—C12	118.2 (4)	O2—C19—C13	119.6 (5)
C1—N1—Mn1	126.1 (3)	O1—C19—C13	115.0 (4)
C12—N1—Mn1	115.3 (3)	N3—C20—C21	122.9 (5)
C10—N2—C11	117.4 (4)	N3—C20—H20	118.5
C10—N2—Mn1	127.5 (3)	C21—C20—H20	118.5
C11—N2—Mn1	114.9 (3)	C22—C21—C20	119.8 (5)
C20—N3—C31	118.5 (4)	C22—C21—H21	120.1
C20—N3—Mn1	124.6 (3)	C20—C21—H21	120.1
C31—N3—Mn1	116.1 (3)	C21—C22—C23	119.6 (5)
C29—N4—C30	117.1 (4)	C21—C22—H22	120.2
C29—N4—Mn1	128.8 (4)	C23—C22—H22	120.2
C30—N4—Mn1	112.7 (3)	C22—C23—C31	117.3 (5)
C19—O1—Mn1	134.6 (3)	C22—C23—C24	124.5 (5)
Mn1—O5—H5A	132.0	C31—C23—C24	118.2 (5)
Mn1—O5—H5B	116.1	C25—C24—C23	122.3 (6)
H5A—O5—H5B	107.2	C25—C24—H24	118.9
H6A—O6—H6B	111.3	C23—C24—H24	118.9
H7A—O7—H7B	111.7	C24—C25—C26	121.7 (5)
H8A—O8—H8B	112.9	C24—C25—H25	119.2
N1—C1—C2	122.6 (5)	C26—C25—H25	119.2
N1—C1—H1	118.7	C27—C26—C30	117.4 (5)
C2—C1—H1	118.7	C27—C26—C25	124.2 (6)
C3—C2—C1	119.9 (5)	C30—C26—C25	118.4 (5)
C3—C2—H2	120.0	C28—C27—C26	120.0 (5)
C1—C2—H2	120.0	C28—C27—H27	120.0
C2—C3—C4	120.1 (5)	C26—C27—H27	120.0
C2—C3—H3	119.9	C27—C28—C29	118.8 (6)
C4—C3—H3	119.9	C27—C28—H28	120.6
C3—C4—C12	116.8 (5)	C29—C28—H28	120.6
C3—C4—C5	124.2 (5)	N4—C29—C28	123.9 (6)
C12—C4—C5	119.0 (4)	N4—C29—H29	118.0
C6—C5—C4	121.0 (5)	C28—C29—H29	118.0
C6—C5—H5	119.5	N4—C30—C26	122.6 (5)
C4—C5—H5	119.5	N4—C30—C31	117.9 (4)
C5—C6—C7	121.6 (5)	C26—C30—C31	119.5 (5)
C5—C6—H6	119.2	N3—C31—C23	121.8 (5)
C7—C6—H6	119.2	N3—C31—C30	118.2 (4)
C11—C7—C8	116.8 (4)	C23—C31—C30	120.0 (5)
C11—C7—C6	119.2 (4)	C33—C32—C37	119.1 (4)
C8—C7—C6	123.9 (5)	C33—C32—C38	120.3 (4)
C9—C8—C7	119.8 (5)	C37—C32—C38	120.5 (5)
C9—C8—H8	120.1	C32—C33—C34	121.2 (5)
C7—C8—H8	120.1	C32—C33—H33	119.4
C8—C9—C10	119.3 (5)	C34—C33—H33	119.4
C8—C9—H9	120.3	C35—C34—C33	117.9 (5)
C10—C9—H9	120.3	C35—C34—H34	121.1
N2—C10—C9	123.3 (5)	C33—C34—H34	121.1
N2—C10—H10	118.4	C34—C35—F2	118.5 (6)
C9—C10—H10	118.4	C34—C35—C36	123.2 (5)
N2—C11—C7	123.3 (4)	F2—C35—C36	118.2 (5)
N2—C11—C12	117.5 (4)	C35—C36—C37	118.5 (5)
C7—C11—C12	119.1 (4)	C35—C36—H36	120.8
N1—C12—C4	122.3 (4)	C37—C36—H36	120.8
N1—C12—C11	117.7 (4)	C36—C37—C32	120.1 (5)
C4—C12—C11	120.0 (4)	C36—C37—H37	120.0
C14—C13—C18	118.5 (4)	C32—C37—H37	120.0
C14—C13—C19	121.4 (4)	O3—C38—O4	126.0 (5)
C18—C13—C19	120.0 (4)	O3—C38—C32	116.3 (5)
C13—C14—C15	122.5 (5)	O4—C38—C32	117.7 (4)

### Hydrogen-bond geometry (Å, °)

**Table:**

*D*—H···*A*	*D*—H	H···*A*	*D*···*A*	*D*—H···*A*
O5—H5A···O3^i^	0.85	1.79	2.622 (4)	166
O5—H5B···O2	0.85	2.06	2.719 (5)	135
O6—H6A···O4^ii^	0.85	1.98	2.825 (6)	171
O6—H6B···O7^iii^	0.85	2.08	2.827 (7)	146
O7—H7A···O8	0.85	2.02	2.854 (8)	165
O7—H7B···O6	0.85	1.99	2.819 (6)	166
O8—H8A···O4	0.85	1.97	2.792 (7)	164
C1—H1···F2^iv^	0.93	2.50	3.209 (7)	133
C5—H5···O3^v^	0.93	2.45	3.339 (7)	160
C20—H20···O4^i^	0.93	2.42	3.233 (7)	146

Symmetry codes: (i) −*x*+1, −*y*+1, −*z*+1;  (ii) −*x*+1, −*y*, −*z*; (iii) −*x*+2, −*y*, −*z*; (iv) −*x*, −*y*+1, −*z*; (v) *x*−1, *y*, *z*.
